# Health care utilization and costs among medical-aid enrollees, the poor not enrolled in medical-aid, and the near poor in South Korea

**DOI:** 10.1186/s12939-015-0257-9

**Published:** 2015-11-14

**Authors:** Jae Woo Choi, Eun-Cheol Park, Sung-Youn Chun, Kyu-Tae Han, Euna Han, Tae Hyun Kim

**Affiliations:** Department of Public Health, Graduate School, Yonsei University, Seoul, Korea; Institute of Health Services Research, Yonsei University College of Medicine, Seoul, Korea; Department of Preventive Medicine, Yonsei University College of Medicine, Seoul, Korea; College of Pharmacy, Yonsei Institute of Pharmaceutical Sciences, Yonsei University, Incheon, Korea; Department of Hospital Administration, Graduate School of Public Health, Yonsei University, 50-1 Yonsei-ro, Seodaemun-gu, Seoul, 120-752 Republic of Korea

**Keywords:** Health care utilization, Medical cost, Economically vulnerable groups

## Abstract

**Background:**

Although government has implemented medical-aid policy that provides assistance to the poor with almost free medical services, there are low-income people who do not receive necessary medical services in Korea. The aim of this study is to highlight the characteristics of Medical-Aid enrollees, the poor not enrolled in Medical-Aid, and the near poor and their utilization and costs for health care.

**Methods:**

This study draws on the 2012 Korea Welfare Panel Study (KOWEPS), a nationally representative dataset. We divided people with income less than 120% of the minimum cost of living (MCL) into three groups (*n* = 2,784): the poor enrolled in Medical-Aid, the poor not enrolled in Medical-Aid (at or below 100% of MCL), and the near poor (100–120% of MCL). Using a cross-sectional design, this study provides an overview of health care utilization and costs of these three groups.

**Results:**

The findings of the study suggest that significantly lower health care utilization was observed for the poor not enrolled in Medical-Aid compared to those enrolled in Medical-Aid. On the other hand, two groups (the poor not enrolled in Medical-Aid, the near poor) had higher health care costs, percentage of medical expenses to income compared to Medical-Aid.

**Conclusion:**

Given the particularly low rate of the population enrolled in Medical-Aid, similarly economically vulnerable groups are more likely to face barriers to needed health services. Meeting the health needs of these groups is an important consideration.

## Introduction

The public healthcare system in Korea has two components, National Health Insurance and Medical-Aid. The national health insurance system, which is managed comprehensively in the form of social insurance, is funded by contributions from beneficiaries and provides coverage to all citizens [1]. The other component – Medical-Aid – is a public medical assistance program targeted at poor individuals who are recipients of the National Basic Livelihood Security System in Korea as a part of the social welfare programs [2, 3]. As of 2012, Medical-Aid had 1,507,044 beneficiaries, representing 3.0 % of the country’s population [4]. The Korean Medical-Aid program is comparable to the US Medicaid program, which was established in 1965, and provided health care services to approximately 58 million people in 2011, including low-income families, seniors, disabled, and pregnant women [5].

Efforts have been made to strengthen the benefit of health care for low-income people in South Korea. Korea’s economic crisis, which occurred in 1997, affected a number of low-income people and limited medical utilization by dropping access to primary care. To solve this problem, Government implemented the Medical-Aid expansion policy in order to increase access to primary care. As a result, the level of preventable hospitalization of those newly enrolled by Medical-Aid decreased significantly compared to those covered by the National Health Insurance [6]. Subsequently, government continuously expanded Medical-Aid subjects in an effort to protect and promote health of those with low-income [7]. In addition, the Ministry of Health and Welfare introduced Medical-Aid for the near poor in 2004; the purpose of this policy was to extend Medical-Aid to the population whose income is more than 100% but less than 120% of the minimum cost of living (MCL), and thus previously had been left in a blind spot [8]. Medical-Aid increased by 15% in 2005 due to policy improvement and, since then, the number of beneficiaries has shown a slight increase. However, patients with a rare and intractable disorder, chronic disease, and children under the age of 18 in Medical-Aid for the near poor were included in National Health Insurance from 2008 after the policy implementation and eligibility transition resulted in a reduction of the number of recipients from 1,852,714 in 2007–1,507,044 in 2012 [9, 4]. In addition, a significant number of low-income persons did not receive Medical-Aid eligibility, even though they are poorer than the near poor. Although their income level is below 100% of the poverty-line, they are excluded when their property is more than criteria or income property of their support obligor is over a certain level [8]. Approximately 10% of the total population of South Korea is below 120% of poverty level; of these, 3.16% are Medical-Aid beneficiaries, and the others are in a blind spot of health care [10].

Even if all Koreans are covered with NHI or Medical-Aid, public expenditure as a proportion of total health expenditure is only 56 %, which is lower than the OECD average (72 %), and is the fourth lowest OECD level of spending after Chile (46 %), USA (48 %), and Mexico (51 %) in 2013 [11]. Previous studies have reported an association of low-income with a greater likelihood of reporting an unmet need for health care [12]. However, no empirical research to elucidate health care utilization and costs among low-income groups divided according to specific criteria has been conducted. Therefore, this study examines general characteristics by dividing Medical-Aid enrollees, the poor not enrolled in Medical-Aid, and the near poor, respectively. In addition, we compare out-of-pocket (OOP) payments, utilization, and the financial burden of health care services among them.

## Methods

### Data

This study used the 2012 Korea Welfare Panel Study (KOWEPS) of Korea, representative national households, conducted by the Korea Institute for Health and Social Affairs along with the Seoul National University Social Welfare Research Center. KOWEPS was collected using an interview research method where the interviewer questioned and recorded the answers of the interviewees. A stratified cluster systematic method was used in selection of the sample of households for the research. This survey was conducted with the goal of understanding the living conditions and welfare demands of the population group, and to evaluate the effectiveness of policies to utilize information of policies. These data are appropriate for analysis of the characteristics of low-income persons, such as Medical-Aid beneficiaries, because more than half of sampling consists of low-income households below 60% of median income and the data include various questionnaires which can be used for in depth examination of utilization of health care. In addition, results of analysis can be easily generalized because KOWEPS has conducted a survey of all people including those in rural areas.

### Study sample

The total sample size of KOWEPS was 17,984 individuals (9,800 households) in 2012. We classified adults under 120% of minimum cost of living, which is measured by the countable income according to three groups (*n* = 2,784). The first group consisted of enrollees in Medical-Aid which did not exceed 100% of poverty (*n* = 1,036). The second group included the poor not enrolled in Medical-Aid which did not exceed 100% of poverty (*n* = 1,325). The third group consisted of adults who were the near poor, ranging from 100–120 % of minimum cost of living (*n* = 523).

### Dependent variable

Three primary dependent variables were examined: out-of-pocket costs, medical utilization, and financial burden of health care services. Out-of-pocket costs were defined as annual average direct payment for hospitalizations, outpatient visits, dental treatment, surgery, prescription drugs, nursing care, and health examination. Medical utilization was measured by the number of outpatient visits and length of stay in hospital per person during a year. Finally, we examined the financial burden of health care services by estimation of the proportion of medical expenses to disposable income [13].

### Explanatory variables

In this study we used several covariates to control for demographic and socioeconomic characteristics and health status. Demographic characteristics included sex, age, marital status, and socioeconomic factors including education and employment status. As a proxy for health status, we used self-rated health, depression, disability, and chronic disease to control for the participant’s health condition and health behavior, which can affect health care utilization.

### Statistical analysis

Data were analyzed using the SAS 9.2 program (SAS Institute, Cary, NC, USA). General characteristics were analyzed using descriptive statistics and comparison between three groups using One-way ANOVA. We analyzed amount of utilization (outpatient visits and length of stay) using negative binomial regression because they are count variables. OOP spending did not show normal distribution, log transformation was performed for the data analyses. Multiple regression method was used to examine the association between explanatory variables and the dependent variables. For all statistical tests, the level of significance was 0.05.

### Definitions

#### Minimum cost of living

Minimum cost of living is minimum expense to sustain one’s life. 2012 minimum monthly cost of living presented by the government was 488 USD (United States Dollars) per single-person household, 831 USD per two-person households, 1,075 USD per three-person households, and 1,319 USD per four-person households. Minimum cost of living is set by rate of increase in a four-person household and is calculated by cost which multiplies the OECD equivalence scale [14].

#### Countable income

Countable income, that is the sum of evaluated income and converted income of property, is used in selection of Medical-Aid enrollees or the near poor compared to minimum cost of living. The evaluated income subtracted from ordinary income to cash benefit is provided by the National Basic Living Security Act and various government subsidies. The converted income of property is the sum of general property, financial property, and converted income for car [15].

## Results

### Baseline characteristics

The demographic, socioeconomic, and health related characteristics of the three groups are shown in Table 1. Compared to Medical-Aid enrollees, the other groups included a higher percentage of persons 65 years and over (48.4% (medical-aid enrollees) versus 81.7% (the poor not enrolled in medical-aid) versus 74.5% (the near poor), *p* < .0001). Medical-Aid enrollees tended to have more education (11.0% versus 5.8% versus 8.3% with above university, *p* < .0001), lower married status (30.5% versus 49.2% versus 48.7%, *p* < .0001), and more disability (33.1% versus 17.1% versus 13.2%, *p* < .0001).

**Table 1:** General characteristics

				Unit: N (%)	
Variables	Total (*N* = 2,784)	Medical-Aid (*N* = 1,036)	Poor not enrolled in Medical-Aid (*N = 1,325*)	The near poor (*N = 523*)	*p*-value
Gender					0.2586
Male	956	375(36.2)	444(33.5)	137(32.4)	
Female	1,828	661(63.8)	881(66.5)	286(67.6)	
Age					<.0001
20 ~ 39	213	124(12.0)	62(4.7)	27(6.4)	
40 ~ 64	672	411(39.7)	180(13.6)	81(19.1)	
≥65	1,899	501(48.4)	1,083(81.7)	315(74.5)	
Education level					<.0001
Below elementary school	1,745	539(52.0)	938(70.8)	268(63.4)	
Middle/high school	813	383(37.0)	310(23.4)	120(28.4)	
Above university	226	114(11.0)	77(5.8)	35(8.3)	
Marital status					<.0001
Married	1,174	316(30.5)	652(49.2)	206(48.7)	
Single	274	186(18.0)	63(4.8)	25(5.9)	
Divorced or separated	1,328	531(51.3)	607(45.8)	190(44.9)	
Employment status					<.0001
Economically active	655	200(19.3)	310(23.4)	145(34.3)	
Inactive/unemployed	2,025	784(75.7)	981(74.0)	260(61.5)	
Self-rated health					<.0001
Good	1,366	468(45.2)	652(49.2)	246(58.2)	
Bad	1,418	568(54.8)	673(50.8)	177(41.8)	
Depression					0.0094
Yes	1,092	423(40.8)	531(40.1)	138(32.6)	
No	1,501	527(50.9)	723(54.6)	251(59.3)	
Disability					<.0001
Yes	625	343(33.1)	226(17.1)	56(13.2)	
No	2,159	693(66.9)	1,099(82.9)	367(86.8)	
Chronic disease					0.2184
Yes	2,303	867(83.7)	1,098(82.9)	338(79.9)	
No	481	169(16.3)	227(17.1)	85(20.1)	
Chronic conditions^§^					
Cancer	76	35(3.4)	28(2.1)	13(3.1)	0.1552
Arthritis, low back pain, disc	644	206(19.9)	348(26.3)	90(21.3)	0.0008
Gastritis, gastric ulcer	62	23(2.2)	31(2.3)	8(1.9)	0.8623
Diabetes mellitus	256	105(10.1)	118(8.9)	33(7.8)	0.3307
Hypertension	573	175(16.9)	298(22.5)	100(23.6)	0.0009
Stroke	112	34(3.3)	60(4.5)	18(4.3)	0.2998
Myocardial infarction	97	30(2.9)	47(3.5)	20(4.7)	0.2199

^§^each rate of chronic conditions is calculated by group

Compared to the near poor, ranging from 100–120 % of minimum cost of living, the other groups (medical-aid enrollees, the poor not enrolled in medical-aid) had a lower percentage of people with inactive/unemployed status (61.5% versus 75.7% versus 74.0%, *p* < .0001), better self-rated health (58.2% versus 45.2% versus 49.2%, *p* < .0001), and a lower percentage had depression (32.6% versus 40.8% versus 40.1%, *p* < .0094). Compared to the other groups, the second group (the poor not enrolled in medical-aid) had more arthritis (26.3% versus 19.9% versus 21.3%, *p* < .0008), however, Medical-Aid enrollees had less hypertension with chronic conditions than the other two groups (16.9% versus 22.5% (the poor not enrolled in medical-aid) versus 23.6% (the near poor), *p* < .0009). No statistically significant differences with regard to sex, presence of chronic disease, and a few chronic conditions (cancer, gastritis, diabetes mellitus, stroke, myocardial infarction) were observed between the three groups.

The average health utilization, out-of-pocket spending, and proportion of medical expenses to income by group without adjusting other covariates are shown in Table 2. Overall Medical-Aid enrollees had more outpatient visits and longer length of stays than both the poor not enrolled in Medical Aid and the near poor. However, the OOP spending and the proportion of medical expenses to income of the Medical-Aid enrollees were lower than those of the poor not enrolled in Medical Aid and the near poor.

**Table 2:** Average health utilization, OOP spending, and proportion of medical expenses to income by group

variables	Medical-Aid		Poor not enrolled in Medical Aid		*p*-value^a^	The near poor		*p*-value^b^
	Mean	Std Dev	Mean	Std Dev		Mean	Std Dev	
The number of outpatient visits	33.8	45.0	30.3	36.6	0.0452	29.5	39.9	0.0668
Length of stay	7.5	26.7	3.6	15.0	<.0001	5.7	19.3	<.0001
OOP spending (unit: USD^§^)	803	172.5	1,226	184.0	<.0001	1,778	232.2	<.0001
Proportion of medical expenses to income (unit: %)	6.5	0.1	16.1	0.8	<.0001	15.8	0.2	<.0001

^a^T-test or Chi-square test results

^b^ANOVA test results

^§^1 USD = 1,134 won

*OOP* out-of-pocket

Table 3 shows multiple regression results after adjusting for socio-demographic characteristics (e.g., age and education) and health status (e.g., self-rated health, chronic disease). The results indicate that compared to the Medical Aid enrollees, only the poor not enrolled in Medical Aid had statistically significantly lower number of outpatient visits and length of stay, but both the poor not enrolled in Medical Aid and the near poor had higher OOP spending as well as percentage of medical expenses to income.

**Table 3:** Factors associated with health utilization, OOP spending, proportion of medical expenses to income

Variables	The number of outpatient visits	Length of stay	Ln (out-of-pocket spending)	% of medical expenses to income
Subject (ref = medical-aid)				
Poor not enrolled in Medical Aid	−0.173***	−0.290**	1.143***	0.090***
The near poor	−0.101	0.243	1.597***	0.109***
Gender (ref = male)				
Female	0.342***	−0.319	0.175*	−0.0223
Age (ref = 20–39)				
40–64	0.204*	0.418	−0.529***	0.012
≥65	0.286**	−0.347	−0.630***	−0.037
Education level (ref = below elementary school)				
Middle/High school	−0.163***	−0.261	0.196**	−0.033
Above university	−0.268**	−0.701	0.480***	−0.109**
Marital status (ref = married)				
Single	−0.038	−1.267**	−0.778***	0.074
Divorced or separated	0.040	0.043	−0.861***	−0.005
Employment status (ref = economically active)				
Inactive/unemployed	0.124**	0.531*	−0.017	0.028
Self-rated health (ref = good)	0.373***	0.944***	0.392***	0.007
Depression (ref = no)	−0.002	0.503**	0.017	0.029
Disability (ref = no)	0.094	0.255	0.103	−0.014
Chronic disease (ref = no)	1.383***	0.954***	0.577***	0.024

^§^Adjusted odds ratio from multiple regression analysis with all of the variables in Table 1

Statistically significant differences are *p < .05 **p < .01 ***p < .001

## Discussion

We performed an analysis by dividing people into three groups, Medical-Aid enrollees, the poor not enrolled in Medical-Aid, and the near poor, and supposed that OOP payment, medical utilization, and financial burden of health care services are significantly different among them. Medical utilization was significantly lower for the poor not enrolled in Medical-Aid compared to Medical-Aid enrollees. These results show that low-income groups excluded in Medical-Aid could have unmet need of medical utilization. This is similar to previous studies that reported higher levels of unmet need and lower rates of health care utilization for low income people compared to middle to high income people [16, 17, 18, 19, 20]. And, indeed, this is borne out, with these US studies identifying uninsured and low income as two of the strongest correlates of unmet need [21–23, 24, 25, 26, 27, 28, 29–31, 32]. In countries with universal health care coverage, such as UK, Germany, and Canada, research on unmet need has been less extensive than in the United States, perhaps because of the relative lack of direct cost-based barriers to care. Studies conducted in Canada have identified some population groups with increased likelihood of reporting an unmet health care need, such as women, people in worse health, nonelderly, higher educated, lower income, nonimmigrants, urban residents, and individuals without prescription drug insurance [33, 34, 35]. In addition, OOP spending of two groups (the poor not enrolled in Medical-Aid, the near poor) was higher than that of Medical-Aid enrollees and proportion of medical expenses to income was significantly higher than that of Medical-Aid enrollees, respectively. This is similar to previous studies that reported higher levels (range from 11.3 to 19.8 %) of proportion of medical expenses to income for low income people compared to middle (range from 3.5–6.6 %) to high (range from 2.0–5.5 %) income people [36, 37].

If we only judge range of subject of application, no one in Korea should be excluded from benefit of medical security. However, there is a blind spot, those who do not receive medical coverage at all between National Health Insurance and Medical-Aid in Korean medical social security. Most of the poor not enrolled in Medical-Aid because of a blind spot are excluded from Medical-Aid due to eligibility of support obligor. Although their income level is lower than minimum of living, they lack the qualifications because income of the support obligor who has responsibility for their support is higher than criteria. Therefore, Government needs to protect people by alleviating the strict criteria of support obligor.

A blind spot in medical security includes the near poor as well as the poor not enrolled in Medical-Aid. Since income of the near poor exceeds a minimum of living, they mainly have unstable employment status such as daily or temporary work. In addition, they have the possibility of losing eligibility to receive social welfare benefits according to longitudinal default of premium or have limited benefit due to relatively low-income level. Actually, the number of low-income people who do not receive benefit of National Health Insurance was 1,170 thousand (4.3% of the total population) in 2011, and this amounts to a third of the poor not enrolled in Medical-Aid [38]. In particular, because the near poor have to pay more out-of-pocket payments than Medical-Aid enrollees who receive benefit of health care, their burden is greater than that of the general population as well as Medical-Aid enrollees. Since South Korea has implemented various medical supporting programs in an effort to solve this problem, most assistance programs support a few non-covered medical costs or partially insured payments for certain diseases such as cancer [39, 19]. Even though they have similar acute or chronic diseases compared to other low-income people, the near poor who have low accessibility have unmet need for health services due to financial burden of health care services, and it could result in reverse discrimination, where their income is lower compared to Medical-Aid enrollees after paying one’s medical costs if demand for health care is high [40, 8]. In other words, since all people excluding Medical-Aid enrollees are National Health Insurance subscribers in the current medical social security, there should be no one without protection. However, the near poor are always in danger of being excluded from protection of medical security until they are able to receive benefit of National Health Insurance through stable income, and fall into medical poverty where they cannot utilize medical services. Such medical poverty could lead to unhealthy status, making one’s work difficult and result in repeat poverty that falls into poverty. Current programs supporting the medical expenses focus on insured payment in South Korea. Since non-insured payment accounted for 16% of total medical cost in 2010 [41], this could result in a heavy burden on the near poor. Thus, government planning for support of increasing financial accessibility of the near poor is needed.

This study has a few limitations. This study was cross-sectional in design; thus, there could be issues of causality. Second, we did not follow up with regard to location of treatment. Delayed reimbursement, a high cutback rate, and the relatively lower profit rate from Medical-Aid patients have led most health care providers to refuse or discriminate against Medical-Aid enrollees [42]. Even though the results will be different according to the hospital type used, we did not identify the information. Third, the prevalence of chronic conditions is likely to be higher than reported in KOWEPS, because some conditions were not diagnosed. Finally, the results of our research are more likely to be statistically biased due to endogeneity problems caused by the selection bias. Since unobserved characteristics would bias the results through error terms or residual, caution is needed in interpreting the results.

Despite these limitations, our research differs from previous studies in dividing income level. Although many previous literature studies regarding exactly the same or similar empirical hypotheses such as medical utilization or expenses of low-income people have already been reported, we used a more delicate design in classifying the subjects for low-income people. In previous studies on this topic in South Korea, the subjects are mainly divided by total income (or disposable income); however, in this study, we used countable income in separating them in this study. As we described above, countable income includes converted income of property as well as evaluated income, and the government mainly utilizes the countable income compared to minimum cost of living when they implement policy according to income level.

## Conclusions

Medical utilization by the poor not enrolled in Medical-Aid and the near poor is significantly lower compared to Medical-Aid enrollees. These results show that low-income groups excluded from Medical-Aid could have unmet need of medical utilization and could experience medical poverty. Therefore, medical benefit for low-income people who are not able to access necessary health care should be intensified. We suggest a few ways to enhance medical benefit for them. First, a premium imposing system in National Health Insurance can transform coinsurance rate into discriminatory imposition according to income level. Second, government should alleviate strict criteria by changing eligibility to receive social welfare benefits and planning for support to increase financial accessibility of the near poor.
